# Extraction Process, Component Analysis, and *In Vitro* Antioxidant, Antibacterial, and Anti-Inflammatory Activities of Total Flavonoid Extracts from *Abutilon theophrasti* Medic. Leaves

**DOI:** 10.1155/2018/3508506

**Published:** 2018-03-13

**Authors:** Chunlian Tian, Peng Zhang, Caixia Yang, Xiang Gao, Hong Wang, Yuru Guo, Mingchun Liu

**Affiliations:** Key Laboratory of Zoonosis of Liaoning Province, College of Animal Science and Veterinary Medicine, Shenyang Agricultural University, No. 120 Dongling Road, Shenhe District, Shenyang, Liaoning Province 110866, China

## Abstract

The flavonoid fraction was extracted from the leaves of *Abutilon theophrasti* Medic., which are usually used as a traditional Chinese herbal medicine for the treatment of inflammation and joint pain. The current study focused on the extraction process, component analysis, and *in vitro* antioxidant, antibacterial, and anti-inflammatory activities of the flavonoid fraction as a part of ongoing research on bioactive substances from natural plant sources. This study evaluated the antioxidant activities via assays of DPPH radical scavenging capacity, ABTS radical scavenging capacity, and reducing power and investigated inhibitory activities against *Escherichia coli*, *Salmonella*, *Staphylococcus aureus*, and *Streptococcus*. Moreover, the inflammatory activity of the flavonoid fraction was estimated by measurement of the content of tumor necrosis factor alpha, interleukin-1-beta, interleukin-6, interleukin-10, nitric oxide, and cyclooxygenase-2 and the gene expression levels of several inflammation markers, such as inducible nitric oxide synthase and cyclooxygenase-2, in RAW 264.7 macrophages after LPS treatment. In addition, the underlying anti-inflammatory mechanisms, that is, the activation of nuclear factor kappa B (NF-*κ*B) and mitogen-activated protein kinase (MAPK) signaling pathways, were also revealed from the gene and protein expression levels. Taken together, these results suggested that the flavonoid fraction might exert *in vitro* antioxidant, antibacterial, and anti-inflammatory effects on LPS-stimulated RAW 264.7 macrophages and will be potentially useful as an adjuvant treatment for oxidative stress and bacterial and inflammatory diseases.

## 1. Introduction

Oxidative stress, which is an important factor that leads to aging and diseases, can be produced by excessive free radicals [1–4]. Therefore, the elimination of harmful free radicals is an effective approach for balancing the internal environment and preventing related diseases. Synthetic antioxidants, such as butylated hydroxytoluene (BHT) and butyl hydroxy anisd (BHA), have been applied widely in the food, pharmaceuticals, cosmetic, and manufacturing industries. However, studies have shown that these antioxidants produce adverse reactions, resulting in diseases and behavioral changes [5, 6].

Furthermore, the body's resistance declines and is accompanied by the various influential factors, including old age, diabetes, liver cirrhosis, cancer, blood diseases, chemotherapy, radiotherapy, immune suppressants, hormones, and antibiotics. Under these circumstances, the body is vulnerable to bacteria, even those that are weakly pathogenic. Therefore, antibiotics are usually a first choice for treatment of infectious diseases induced by bacterial or pathogenic microorganisms. However, their adverse effects, such as anaphylaxis [7, 8], intestinal flora alteration [9, 10], and, in particular, drug resistance [11, 12], are often overlooked.

In addition, inflammation, which is a major defense reaction of the immune system [13] to harmful stimuli, including infection and injury, is a serious threat to health [14, 15] and exists in many diseases, such as bronchitis, pneumonia, gastritis, nephritis, and rheumatism. At present, steroidal and nonsteroidal anti-inflammatory drugs are commonly used in clinics; nevertheless, their side effects, such as gastrointestinal tract damage [16, 17] and allergic reactions [18–20], cannot be ignored.

Briefly, oxidative damage, bacterial infection, and inflammation are relatively typical clinical symptoms in many diseases. However, increasing attention has been given to adverse drug reactions. Therefore, it is necessary to carry out research for the development of natural, green pharmaceuticals.

*Abutilon theophrasti* Medic. (*A. theophrasti*) is grown widely in the tropics and subtropics, such as China, Vietnam, India, Japan, Europe, and North America [21], and is rich in potentially important bioactive compounds, including flavonoids, phenolics, organic acids, polysaccharides, saponins, amino acids, and coumarin [22–24]. Moreover, this plant has been utilized in folk treatments for ulcers, swell, venom, inflammation, and pain [25–27]. However, the antioxidant, antibacterial, and anti-inflammatory activities of *A. theophrasti* leaves have not been extensively investigated.

Preliminary studies showed that *A. theophrasti* roots, stems, leaves, seeds, and episperm are rich in flavonoids and that the leaves contain the highest content of total flavonoids [24]. It is well known that flavonoids have a wide range of biological activities and pharmacological effects, such as antioxidant, antibacterial, anti-inflammatory, anticancer, and antiviral activities and effects [28–33]. To evaluate the therapeutic potential of the flavonoid fractions of medicinal herbs, *A. theophrasti* leaves, traditionally used in China, were studied for their antioxidant, antimicrobial, and anti-inflammatory activities against free radicals, pathogenic bacteria, and inflammation induced by LPS in RAW 264.7 cells. Moreover, anti-inflammatory mechanisms were further revealed by analysis of the expression of genes and proteins that are associated with nuclear factor kappa B (NF-*κ*B) and mitogen-activated protein kinase (MAPK) signaling pathways.

Therefore, the objectives of this paper were (a) to investigate the process of extracting the flavonoid fraction of *A. theophrasti* leaves by single-factor experiments and response surface method; (b) to identify the chemical components in the extract by HPLC-DAD-ESI-MS^n^; (c) to evaluate the *in vitro* antioxidant, antibacterial, and anti-inflammatory activities of the total flavonoid extract (TFE); and (d) to analyze the anti-inflammatory mechanisms that are associated with the NF-*κ*B and MAPK signaling pathways.

## 2. Materials and Methods

### 2.1. Plant Collection

*A. theophrasti* leaves were collected from Jilin Province of China in August, September, and October (numbers 1408, 1409, and 1410, resp.) and were authenticated by Professor Jingming Jia of Shenyang Pharmaceutical University according to the Compendium of Materia Medica. The voucher specimens KT/JL/CH/AT/08/14, KT/JL/CH/AT/09/14, and KT/JL/CH/AT/10/14 were deposited at our laboratory for future reference. The leaves were washed and dried in a shady and dry place at room temperature for 30 days and were then ground to a fine powder before extraction.

### 2.2. Chemicals and Reagents

2,2′-Azino-bis(3-ethylbenzothiazoline-6-sulfonic acid diammonium salt) (ABTS), 2,2-diphenyl-1-picrylhydrazyl (DPPH), and 2,4,6-tri(2-pyridyl)-*s*-triazine (TPTZ) were purchased from Sigma-Aldrich Chemie (Steinheim, Germany). *Escherichia coli* lipopolysaccharide (LPS), dimethyl sulfoxide (DMSO), and 3-(4,5-dimethylthiazol-2-yl)-2,5-diphenyltetrazolium bromide (MTT) were purchased from Sigma-Aldrich (St. Louis, MO, USA). Interleukin-1 beta (IL-1*β*), IL-6, IL-10, and tumor necrosis factor alpha (TNF-*α*) Elisa kits were obtained from eBioscience (Science Center Drive San Diego, CA, USA). COX-2 Elisa kit was purchased from Axygen (Central Avenue Union City, CA, USA). Antibodies against I*κ*B*α*, p-I*κ*B*α*, NF-*κ*B p65, p-NF-*κ*B p65, p38*α*, p-p38*α*, JNK1/2, p-JNK1/2, ERK1/2, p-ERK1/2, and *β*-actin were purchased from Santa Cruz Biotechnology (Santa Cruz, CA, USA), and secondary antibodies were obtained from Sangon Biotech (Shanghai, China). Other chemicals and reagents were all analytically pure and obtained from Sinopharm Chemical Reagent Co. Ltd. (Shanghai, China).

### 2.3. Instruments

LC-MS/MS analysis was performed on an Agilent 1100 series HPLC (Palo Alto, CA, USA) equipped with a G1311A quaternary pump, a G1313A high-performance autosampler, a G1379A in-line degasser, and a G1316A thermostatted column compartment. The mass spectrometer was an Agilent Ion Trap MSD SL instrument equipped with an atmosphere pressure chemical ionization (APCI) source coupled with an electrospray ionization (ESI) interface. Instrument operation, data acquisition, and processing were performed by MassHunter Workstation software (version B.03.01, Agilent). Chromatograph separation was carried out by liquid chromatography coupled to diode array detection (LC-DAD) analysis. UV spectra were recorded in a range of 210–400 nm and monitored at 350 nm.

### 2.4. Preparation of Extract

A suitable amount of *A. theophrasti* leaf powder was accurately weighed and extracted with a 42.28% ethanol solution, a 30 : 1 (ml/g) ratio of solvent to material, and an ultrasonic-assisted extraction time of 30 min at room temperature. The extraction solution was first filtered through quantitative filter paper, and the medicinal material residue was further extracted one time by using the same extraction solvent, ratio of solvent to material, and ultrasonic-assisted extraction time. The two filtrates were pooled together and then evaporated to yield a solid residue by rotary evaporator. The solid residue was dissolved with a proper solvent and stored in a refrigerator (4°C) until used for analysis.

### 2.5. Total Flavonoid Content

Total flavonoid content (TFC) was measured by a colorimetric method [34] with minor modifications, and rutin was used as a standard. The chromogenic reaction system consisted of aluminum nitrate, sodium nitrite, and sodium hydroxide. A total of 5 ml of extract solution (0.05 g/ml) was added in a 10 ml volumetric flask and then mixed with 0.3 ml of 5% sodium nitrite solution. After reaction for 6 min at room temperature, 0.3 ml of 10% aluminum nitrate solution was added, and then, 4 ml of 4% sodium hydroxide solution was added after reacting for another 6 min. Finally, the volume of the reaction solution was increased to 10 ml with ethanol. The reaction solution was mixed thoroughly and incubated for 20 min at room temperature. After high-speed centrifugation for 6 min, the absorbance of the supernatant was measured at 510 nm. TFC was defined as follows: extraction yield of total flavonoids (*w*/*w*) (%) = mass of total flavonoids expressed as mg of rutin equivalents per g dry weight of *A. theophrasti* leaf sample (mg RE/g DW). All tests were performed in triplicate, and the means were calculated.

### 2.6. Flavonoids Compositions Analyzed by HPLC-MS

#### 2.6.1. Sample Preparation for HPLC-MS Analysis

A total of 0.5 g of *A. theophrasti* leaf powder was extracted with the optimum extraction conditions and dried by vacuum rotary evaporation to yield a solid residue. The solid residue was dissolved with 10 ml of chromatographic methanol (Merck Serono Co. Ltd., Germany), centrifuged (2000 rmp), and filtered with a 0.22 *μ*m microfiltration membrane prior to LC-DAD and LC-MS analysis.

#### 2.6.2. LC-MS/MS and LC-DAD Conditions and Parameters

The molecules were separated on a C_18_ column (Dikma platinum series, 250 mm × 4.6 mm, i.d. 5 *μ*m) using the following mobile phase: (A) acetonitrile and (B) water formic acid 0.1%. LC separation conditions consisted of an isocratic step at 0–45% of A for 0–45 min and then 45–0% of A for 45–50 min, with a flow rate of 700 *μ*l/min. The HPLC-ESI-MS/MS operating conditions were as follows: negative and positive ion modes, automatic secondary mass spectrum scan with a scanning range of 50–1000 m/z, drying gas with 12 l/min and 350°C, nebulizer pressure of 30 psi, and a capillary voltage of 3500 V.

For LC-DAD analysis, the same chromatographic conditions described for LC-MS/MS analysis were employed, with the exception of an injection volume of 20 *μ*l.

### 2.7. Determination of Chemical-Based Antioxidant Activities

#### 2.7.1. ABTS Radical Scavenging Activity Assay

The ABTS radical scavenging activity of TFE from *A. theophrasti* leaves was assayed by the method of Dawidowicz and Olszowy [35] with minor modifications. The ABTS radical cation (ABTS^+^) was obtained in a reaction of 7 mmol/l ABTS and 2.45 mmol/l potassium persulfate. After the mixture was incubated at room temperature in the dark for 12–16 h, the ABTS solution was diluted with PBS to obtain an absorbance of 0.700 ± 0.02 at 734 nm. A total of 0.1 ml of different concentrations of TFE was mixed with 0.9 ml of the ABTS solution, and the absorbance of the resulting solution was measured at 734 nm after a 30 min incubation period at room temperature in a water bath in the dark. The scavenging effect was calculated by the following equation: radical scavenging rate (%) = [(*A*_0_ − *A*_1_)/*A*_blank_] × 100%, where *A*_0_ and *A*_1_ are the absorbance of the ABTS solution and ABTS solution with sample at different concentrations, respectively. The antioxidant activity was expressed as the IC_50_ (mg/ml), which is the TFE concentration that inhibits 50% of the free radicals.

#### 2.7.2. DPPH Radical Scavenging Activity Assay

The DPPH· radical scavenging activity of TFE was measured by the method described by [36] with modifications. A total of 0.1 ml of different concentrations of sample was mixed with 0.9 ml of 0.1 mmol/l DPPH radical ethanol solution. The mixture was shaken and incubated for 30 min at 37°C in a water bath in the dark, and the absorbance of the resulting solution was assayed at 517 nm. The values of IC_50_ were determined as reported above.

#### 2.7.3. Ferric-Ion Reducing Antioxidant Power Assay

The ferric-ion reducing antioxidant power (FRAP) of TFE was measured by the method of Pajak et al. [37] with modifications. Briefly, the FRAP reagent was a mixture of acetate buffer (pH 3.6), 20 mmol/l FeCl_3_, and 10 mmol/l TPTZ in 40 mmol/l HCl at 10 : 1 : 1 (*v*/*v*/*v*). An aliquot of 0.1 ml of different concentrations of TFE was added to 0.4 ml of FRAP reagent, the solution was incubated in the dark for 30 min at 37°C in a water bath, and the absorbance was measured at 593 nm. The values were expressed as mmol Fe^2+^ per 100 *μ*g/ml of TFE. All determinations were performed in triplicate.

### 2.8. Determination of Antimicrobial Activity

#### 2.8.1. Microbial Strains

The standard strains of *Escherichia coli* (ATCC 25922), *Salmonella typhimurium* (ATCC 51812), *Staphylococcus aureus* (ATCC 25923), and *Streptococcus pneumoniae* (ATCC 49619) were obtained from American Type Culture Collection and stored at the Department of Animal Pharmacy, College of Animal Science and Veterinary Medicine, Shenyang Agricultural University.

#### 2.8.2. Bacterium Solution Preparation

*Escherichia coli*, *Salmonella typhimurium*, *Staphylococcus aureus*, and *Streptococcus pneumoniae* were inoculated onto MacConkey agar medium, SS agar medium, hypersalt mannitol medium, and improved Edward medium, respectively. For *Streptococcus*, rabbit blood (without fibrin) was added to the culture medium to produce a final concentration of 5%. After overnight culture, the four standard strains were adjusted to 0.5 McFarland turbidity standards (1 × 10^8^ CFU/ml) and were then diluted to a ratio of 1 : 1000 with sterile nutrient broth.

#### 2.8.3. Sample Solution Preparation for Antibacterial Activity Assay

TFE was prepared with optimal extraction technology and was dissolved in sterile water to obtain an initial stock solution concentration of 2.0 g of raw medicinal material/ml. Ten serial twofold dilutions of the stock solution were prepared to produce a final concentration of 1.0 to 0.002 g of raw medicinal material/ml. The stock solution concentration of gentamicin was 1.28 mg/ml, and the final concentration ranged from 0.25 to 128 *μ*g/ml.

#### 2.8.4. Antibacterial Activity Assay

Antibacterial activity was screened by the microwell dilution method as described by Jaradat et al. [38] with modifications, and the MIC values, defined as the lowest concentration of samples showing clear wells or with complete inhibition of bacteria, were chosen as the primary evaluation indicators. Briefly, each well of a 96-well plate was filled with 100 *μ*l TFE solution and 100 *μ*l of bacterium solution and was completely mixed. The plates were covered and incubated at 37°C for 24 h. The antibiotic gentamicin and sterile water (in a similar volume as the test sample) were used as the positive and negative controls, respectively. Each experiment was performed in triplicate.

### 2.9. Determination of Anti-Inflammatory Activity Assay

#### 2.9.1. Cell Culture

RAW 264.7 cells belonging to a murine macrophage cell line were purchased from the Shanghai Cell Bank of the Chinese Academy of Sciences (Shanghai, China). These cells were cultured in RPMI 1640 medium containing 10% fetal bovine serum, 100 U/ml penicillin, and a streptomycin mixture with supplemented 5% CO_2_ at 37°C.

#### 2.9.2. Sample Solution Preparation for Anti-Inflammatory Activity

TFE solution was prepared with the optimized extraction process and was dissolved completely with RPMI 1640 medium at a final concentration of 13.14 mg/ml. Then, TFE solution was filtered, packed, and preserved at −20°C for later use.

#### 2.9.3. Cell Viability MTT Assays

The cytotoxicity of TFE in RAW 264.7 cells was evaluated using the MTT assay based on the conversion of MTT into formazan crystals in living cells [39]. In brief, cells were cultured in 96-well plates with 4 × 10^4^ cells/well until confluent. After one hour incubation, the cells were grouped into a negative control group, an LPS model group, and three test groups with final TFE concentrations of 50, 100, and 200 *μ*g/ml, respectively. Then, 50 *μ*l of LPS (1 *μ*g/ml) was added to the LPS model group and three test groups, and 50 *μ*l of RPMI 1640 medium was added to the negative control group. After all groups were incubated for 18 h, 20 *μ*l of MTT reagent (5 mg/ml) was added, and each well was incubated for 4 h. Then, 150 *μ*l of dimethyl sulfoxide was mixed after discarding the supernatant and shaken gently, followed by microplate reader detection at 570 nm. Data are representative of three independent assays.

#### 2.9.4. Measurement of COX-2 and NO

In total, 4 × 10^5^ cells were seeded into 96-well plates. After incubation for 1 h, 50 *μ*l of LPS (1 *μ*g/ml) was added to the LPS model group and three test groups, and 50 *μ*l of RPMI 1640 medium was added to the negative control group. The supernatant was collected for measurement of COX-2 and NO after treatment for 18 h.

*(1) COX-2 Measurement*. The content of COX-2 in the culture supernatant was quantified using an ELISA kit in accordance with the manufacturer's instructions. The OD values were measured at 450 nm. After stop buffer is added, quantification must be completed within 15 min.

*(2) NO Measurement*. To prepare solvent A, 1.0 g of anhydrous sulfanilic acid, 6 ml of 85% phosphoric acid, and 70 ml of deionized water were mixed and dissolved adequately, and then, the mixture was diluted to 100 ml in a volumetric flask.

To prepare solvent B, 0.1 g of *n*-naphthylethylenediamine was dissolved fully in deionized water and diluted to a volume of 100 ml.

The NO content was assayed by the Griess reagent method with modifications described by Su et al. [40]. Briefly, 50 ml of different concentrations of sodium nitrite standard diluents and the supernatant was reacted with 50 ml of solvent A and incubated at 37°C for 10 min. Then, 50 ml of solvent B was added, and the mixture was incubated again at 37°C for 10 min. After incubation, the absorbance was measured at 540 nm. The concentration of NO was calculated from an established sodium nitrite standard curve. The data are representative of three independent experiments.

#### 2.9.5. Measurement of Cytokine Secretion

The proinflammatory cytokines TNF-*α*, IL-1*β*, and IL-6 and the anti-inflammatory cytokine IL-10 from the supernatant of RAW 264.7 cell cultures were assayed by ELISA kits according to the manufacturer's instructions. Briefly, 100 *μ*l of diluted capture antibody was added to each well and incubated overnight at 4°C. After the supernatant was discarded, the wells were washed 4 times with a special ELISA lotion. For each wash, the wells were soaked for approximately 1 min, and the remaining fluid was subsequently removed with blotting paper. Next, 200 *μ*l of sample diluent was added to each well, and the plate was sealed with single-use sealing membrane and incubated for 1 h at 37°C. Then, 100 *μ*l of eight different concentrations of standards and culture supernatant was added to each well and mixed with 100 ml of diluted detection antibody, and the plate was then incubated for 1 h at 37°C. After washing, 100 *μ*l of horseradish peroxidase was added, and the plate was sealed for a 30 min incubation. Then, 100 *μ*l of tetramethylbenzidine color developing agent was added and incubated for 15 min. Finally, every well was reacted with 50 *μ*l of stop buffer, and absorbance was measured at 450 nm using a microplate reader. The cytokine concentrations were calculated from a standard curve on the basis of absorbance values.

#### 2.9.6. Total RNA Extraction and Gene Expression Level Assay by Quantitative Real-Time PCR

Total RNA of RAW 264.7 cells was extracted with TRIzol reagent (Vazyme Biotech Co. Ltd., USA) according to the manufacturer's instructions, and gene expression levels were quantified by quantitative real-time polymerase chain reaction (qRT-PCR) as described previously [41]. PCR products were analyzed by a spectrophotometer (DU 730, Beckman Coulter, USA) and 1% agarose gel electrophoresis for quality control. The expression level of mRNA was analyzed using an IQTM5 Optical Module fluorescence quantitative gene amplification instrument (Bio-Rad, USA). RNA was reverse-transcribed into cDNA according to the operating instructions of the reverse transcription reaction system and was used as a template for amplification by PCR. The fluorescence qRT-PCR primer sequences (sequence 5′–3′) are shown in Table 1. After normalization with *β-actin* (internal control), fold changes in the expression levels of target mRNAs (*COX-2*, *iNOS*, *IκB*, *p65*, *p38α*, *JNK*, *a*nd *ERK1/2*) were calculated using the 2^−△△CT^ method.

#### 2.9.7. Protein Extraction and Western Blot Analysis

RAW 264.7 cells and test samples (final TFE concentrations of 50, 100, and 200 *μ*g/ml) were incubated with RPMI 1640 medium for 1 h and then induced with LPS (1 *μ*g/ml) for 30 min. After the supernatant was discarded, the cells were washed twice with precooled PBS and then disrupted with a mixture of cell lysis buffer and phenylmethanesulfonyl fluoride (*v* : *v*, 9 : 1; 1.0 ml per well) for 2-3 h at 4°C for the extraction of total proteins. Cell lysates were centrifuged at 14,000 ×g for 15 min at 4°C, and then, the supernatant was collected for further analysis. The total protein concentration was measured by a BCA protein assay kit (CWBIO Biotechnology Co. Ltd., Beijing, China) according to the manufacturer's instructions.

The protein samples were separated by sodium dodecyl sulfate-polyacrylamide gel electrophoresis (SDS-PAGE) and transferred to polyvinylidene fluoride (PVDF) membranes (Solarbio Technology Ltd., Beijing, China). The PVDF membranes were blocked with 5% skimmed milk in TBST buffer containing 0.05% Tween 20 for 1 h and then incubated for 2 h at 37°C. Subsequently, the membranes were incubated sequentially at 4°C overnight with primary monoclonal antibodies, including I*κ*B*α*, p-I*κ*B*α*, NF-*κ*B p65, p-NF-*κ*B p65, p38*α*, p-p38*α*, JNK1/2, p-JNK1/2, ERK1/2, p-ERK1/2, and *β*-actin (Santa Cruz Biotechnology Co. Ltd., USA). After washing 3–5 times, the membranes were incubated continuously with secondary antibodies labeled with horseradish peroxidase for 2 h at 37°C. Finally, chemiluminescence detection was conducted using an ECL assay kit (Beyotime Biotechnology Co. Ltd., Shanghai, China), and the images were captured using a Micro Chemic Gel Doc XR (Bio-Rad, USA). The relative levels of target proteins were determined based on the optical density of the electrophoresis bands, with *β*-actin serving as an internal control.

### 2.10. Statistical Analysis

Experiment values are means of three replicate samples (*n* = 3). All experiment results were expressed as means ± standard deviation. The RSM experiment data were analyzed by Design Expert 8.0.6 software, and other experiment data were analyzed statistically by SPSS 17.0 (SPSS 17.0 for Windows; SPSS Inc., Chicago, IL). In all statistical analyses, *p* values ≤ 0.05 were regarded as statistically significant and *p* values ≤ 0.01 as very significance.

## 3. Results and Discussion

### 3.1. Optimize Extraction Conditions for Total Flavonoids

#### 3.1.1. Experimental Design

*(1) Single Factor Experiments*. According to related literature [42–44] and preliminary single-factor and response surface experiments, three major influence factors, namely, the extraction time (*A*, min), concentration of ethanol solution (*B*, %), and ratio of solvent to material (*C*, ml/g), were investigated for optimum extraction, and the yields of the flavonoid fraction from *A. theophrasti* leaves were determined using an inspection index. The flavonoid fractions were prepared on the basis of the following factors: concentration of ethanol solution (20%, 40%, 60%, 80%, and 100%), extraction time (10, 15, 20, 25, and 30 min), and ratio of solvent to material (10 : 1, 15 : 1, 20 : 1, 25 : 1, and 30 : 1 ml/g) in two extraction cycles. As one influence factor was evaluated, the middle levels were chosen for the other two factors. The filtrate was concentrated to 10 ml prior to the measurement of TFC.

*(2) Optimization of Extraction Conditions by a Box-Behnken Design*. On the basis of the single-factor experiments, three levels were confirmed for the three factors, and then, a Box-Behnken design (BBD) of three variables (*A*, extraction time; *B*, concentration of ethanol solution; and *C*, ratio of solvent to material) and three levels (Design-Expert software, Trial Version 8.0.6, State Inc., Minneapolis, USA) was introduced to optimize the extraction procedure of the flavonoid fraction from *A. theophrasti* leaves. The independent variables and the ranges of their levels are listed in Table 2, and the yield of the flavonoid fraction was considered the dependent variable. As shown in Table 3, the BBD consisted of twelve factorial points and five replicates of central points in the response surface experiment, and the experimental runs were randomized to minimize the effects of unpredictable variability in the observed responses.

#### 3.1.2. Optimization of Extraction Conditions by BBD

*(1) Model Fitting and Statistical Analysis*. As shown in the design matrix (Table 3), 17 experiments were performed with the yield of the flavonoid fraction from *A. theophrasti* leaves as an index. The data were analyzed statistically by multiple regression analysis using Design Expert software (version 8.0.6) to obtain the following flavonoid fraction final equation in terms of coded factors:

(1) $$\begin{array}{c} Y=1.09+0.074A-0.01B+0.061C+0.088AB-0.005AC-0.047BC+0.002A^{2}-0.066B^{2}+0.072\;C^{2}. \end{array}$$

According to the calculated coefficient (*R*^2^ = 0.9974) obtained by ANOVA of the quadratic regression model (Table 4), only 0.26% of total variance cannot be explained by the model, and the adjusted *R*^2^ at 0.9941 demonstrated a better correlation between the experimental values and predicted values. In addition, the lower *p* value (*p* ≤ 0.0001) suggested that the model significantly represents the true relationship between the parameters and the response. Furthermore, a lower coefficient of variation (CV) of 0.69 indicates the better precision, accuracy, reliability, and reproducibility of the model.

ANOVA was applied to evaluate the significance of the regression coefficients of the response surface quadratic regression model. As shown in Table 4, the linear coefficients and quadratic term coefficients of the extraction time (*A*), concentration of ethanol solution (*B*), and ratio of solvent to material (*C*) were all very significant (*p* ≤ 0.01). Furthermore, there were very significant differences (*p* ≤ 0.01) in the interactions between *A* and *B* and between *B* and *C*, while no significant linear and quadratic effect was observed between *A* and *C* (*p* > 0.05). In addition, *B*^2^ and *C*^2^ had a very significant (*p* ≤ 0.01) influence on the yield of the flavonoid fraction, which indicated that the influence factors did not have a simple linear or quadratic relationship (Table 4).

*(2) Graphical Interpretation and Optimization of Procedure*. The relationship between the response and the experimental level for each of the three factors was visualized by the surface and contour plots on the basis of the derived equations, and the optimum conditions for the maximum yield of TFE could be deduced.

**Figure 1:**
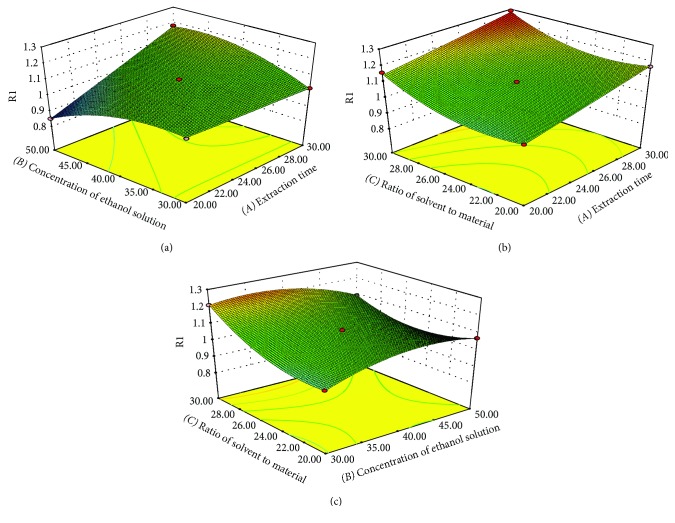
(a–c) Response surface plots showing the effect of extraction time (*A*, min), concentration of ethanol solution (*B*, %) and ratio of solvent to material (*C*, ml/g) on TFE yield.

As shown in Figure 1, the extraction yield of TFE from *A. theophrasti* leaves can also be predicted by the three-dimensional (3D) response surface and two-dimensional (2D) contour plots of extraction time (*A*), concentration of ethanol solution (*B*), and ratio of solvent to material (*C*), which influence the yield of TFE to with different degrees.

Figure 1(a) shows that the interaction between the extraction time (*A*) and concentration of ethanol solution (*B*) had a very significant influence (*p* ≤ 0.01) on the yield of TFE when the ratio of solvent to material (*C*) was kept at a middle level. The experimental results demonstrated that the yield of TFE increased when the extraction time was increased from 20 to 30 min. However, compared to the growth rate increase from 20 to 25 min, the extraction yield increased slowly from 25 to 30 min. In consideration of the time, cost, and test index, 30 min was set as the longest extraction time.

The extraction time (*A*) and the ratio of solvent to material (*C*) had minor effects on the TFE yield (Figure 1(b)). The extraction yield increased with changes in the extraction time (*A*) from 20 to 30 min and in the ratio of solvent to material from 20 to 30 ml/g. However, the interaction effect for the yield of TFE relative to the extraction time and to the ratio of solvent to material was not significant (*p* > 0.05).

The extraction yield gradually increased with increasing concentrations of ethanol solution reaching a maximum at 42.8% and finally declined within the range of 42.8 to 50% (Figure 1(c)). However, the yield was positively correlated with the increasing ratio of solvent to material from 20 to 30 ml/g. The mutual interaction between the ethanol concentration and the ratio of solvent to material was very significant (*p* ≤ 0.01). The above results were in accordance with the ANOVA test.

*(3) Optimization of Extraction Parameters*. Because of the optimum extraction conditions that were visually observed, the extraction processing parameters were optimized by a response surface methodology instead of by the orthogonal test (60% ethanol solution, ratio of solvent to material 30 : 1 (ml/g), and extraction time 20 min) that was published previously [45]. The processing parameters were optimized by Design-Expert software as follows: extraction time 30 min; concentration of ethanol solution 42.28%; and ratio of solvent to material 30 : 1. For operational convenience, the concentration of ethanol solution was adjusted to 42.3%. The experiments were performed in triplicate under the revised conditions, and the yield (1.31 ± 0.01%) was only slightly higher than the predicted result (1.29%), indicating a good correspondence between the experimental and predicated values.

#### 3.1.3. TFC of *A. theophrasti* Leaves

The flavonoid fraction from *A. theophrasti* leaves was extracted by conditions that were optimized with model equations, and the yields of the flavonoid fractions were 1.29 ± 0.02%, 1.31 ± 0.01%, and 0.83 ± 0.02% for the samples collected in August, September, and October 2014, respectively (Table 5). The results indicated that there was a clear correlation between TFC and the month in which the medicinal materials were harvested. The order of TFC from *A. theophrasti* leaves in different harvest months was as follows: September > August > October. This finding suggests that TFC might be related to the external environment and internal physiological factors. Therefore, it is necessary to choose an appropriate time for collecting medicinal materials to be used for research on the pharmacological action and content of active components of TFC.

### 3.2. Chemical Component Analysis of TFE

**Figure 2:**
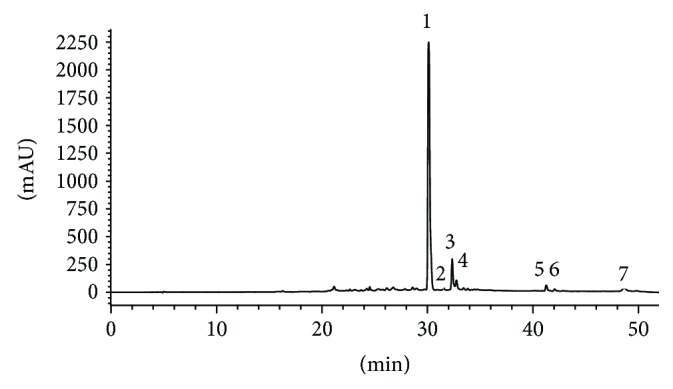
Chromatographic profile at 350 nm of TFE of *A. theophrasti* leaves.

The chemical components present in TFE were identified and analyzed by HPLC-DAD-ESI-MS^n^ in positive and negative ion modes. The UV chromatogram was recorded at 350 nm (Figure 2) and exhibited peaks of multiple heights. The mass and MS/MS fragmentation patterns obtained from the electrospray mass spectra are shown in Table 6. Using this procedure, rutin, quercetin 7-*O*-*β*-glucoside, kaempferol 3-*O*-*α*-rhamnopyranosyl(1→6)-*β*-glucopyranoside, luteolin, apigenin 7-*O*-*β*-diglucoside, poncirin, and tiliroside were characterized tentatively according to the literature [24, 46–49]. This research will provide a foundation for the study of the quality control methods and pharmacological effects of TFE from *A. theophrasti* leaves.

### 3.3. Antioxidant Activity Analysis

As described in Table 5, the highest antioxidant activity was measured in the Sep. sample, which had the lowest IC_50 ABTS_ (2.95 *μ*g/ml) and IC_50 DPPH_ (8.41 *μ*g/ml) and the highest EC_50 FRAP_ (0.41 mmol Fe^2+^/0.1 *μ*g/ml); followed by the Aug. sample, with IC_50 ABTS_ = 3.10 *μ*g/ml, IC_50 DPPH_ = 8.96 *μ*g/ml, and EC_50 FRAP_ = 0.33 mmol Fe^2+^/0.1 *μ*g/ml; and finally Oct. sample, with IC_50 ABTS_ = 5.31 *μ*g/ml, IC_50 DPPH_ = 14.41 *μ*g/ml, and EC_50 FRAP_ = 0.23 mmol Fe^2+^/0.1 *μ*g/ml. Compared to the BHT positive control, with IC_50 ABTS_ 2.25 *μ*g/ml, IC_50 DPPH_ 8.11 *μ*g/ml, and EC_50 FRAP_ 0.14 mmol Fe^2+^/0.1 *μ*g/ml, the Sep. sample displayed much higher FRAP and nearly identical scavenging abilities for DPPH and ABTS free radicals. Furthermore, the FRAPs of the Aug. and Oct. samples were approximately 2.4-fold and 1.7-fold stronger than BHT, respectively.

The three antioxidant assays suggested that the antioxidant activities of TFE correlated with the months during which *A. theophrasti* leaves were collected. Because of their reducing power, all three samples can replace synthetic antioxidant BHT in the pharmaceutical and food industries. In view of the scavenging abilities of the DPPH and ABTS free radicals, the antioxidant activities of the Aug. and Sep. samples were higher than those of the Oct. sample. Therefore, they are likely to be able to substitute for synthetic antioxidants. Therefore, *A. theophrasti* leaves should be a good candidate for the research and development of healthy and natural antioxidants in the food and pharmaceutical industries.

### 3.4. Pearson Correlation Analysis between TFC and Antioxidant Activity

In this research, the antioxidant activities of TFE were investigated using assays of ABTS radical scavenging activity, DPPH radical scavenging activity, and FRAP. To further understand the interrelationships between the antioxidant activity and TFC, the Pearson test was used to analyze the interaction between the two factors, and the results of the correlations among the parameters are shown in Table 7. The correlations were significant (*p* ≤ 0.05) between TFC and the antioxidant activity measured by the ABTS assay (*r*, −1.000) and between TFC and the results of the DPPH assay (*r*, −0.999). Furthermore, there was a better correlation (*r*, 0.912) between TFC and the values of the FRAP assay, although the correlation was not significant (*p* > 0.05). These results indicated that TFC contributes to the antioxidant activity and that the correlation is higher between the antioxidant activity and TFC.

Table 7 also shows the results of correlations between the three methods used to evaluate the antioxidant activity of TFE. A significant correlation (*p* ≤ 0.05) was found between the DPPH and ABTS assays (*r*, 1.000). Moreover, better correlations were found between FRAP and ABTS and FRAP and DPPH, with *r* values of −0.920 and −0.930, respectively. This implies that the correlations were much better for the three quantification methods of antioxidation activity of TFE, and the results were consistent with those of the three antioxidant assays.

### 3.5. Antimicrobial Activity Analysis

The antibacterial activities of TFE from *A. theophrasti* leaves against four bacteria strains are shown in Table 8. Gentamicin, as a standard antibiotic, was used as a positive control to ensure the accuracy and reliability of the assay method for antibacterial activity. As shown in Table 8, the order of the inhibitory effect of TFE on the four bacteria was as follows: first, *Staphylococcus aureus*, with an MIC value of 0.06 ± 0.01 g crude drug/ml; second, *Streptococcus*, with an MIC value of 0.26 ± 0.01 g crude drug/ml; third, *Salmonella*, with an MIC value of 0.51 ± 0.02 g crude drug/ml; and finally, *Escherichia coli*, with an MIC value of 1.02 ± 0.04 g crude drug/ml. These results suggested that the inhibitory effect of TFE on the four bacteria is greater than that of gentamicin. In addition, the inhibitory effect of TFE was stronger on the gram-positive bacteria than on the gram-negative bacteria. This study confirmed that TFE exhibits potential antibacterial activity and provides a foundation for research on replacing or decreasing antibiotic applications in the clinic.

### 3.6. Anti-Inflammatory Activity Analysis

#### 3.6.1. Effect of TFE on the Cell Viability

**Figure 3:**
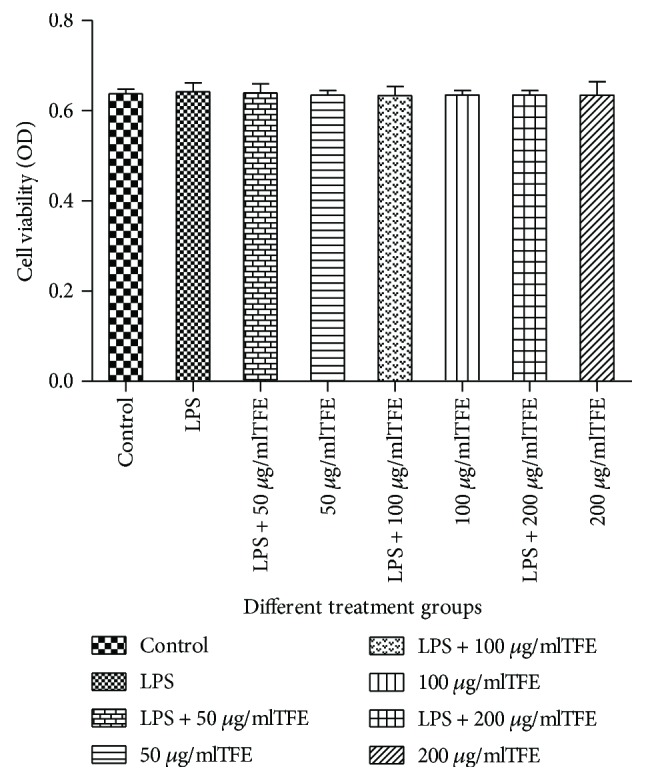
Effect of TFE on the viability of RAW 264.7 cells. Cells were cultured with TFE (50, 100, and 200 *μ*g/ml) in the absence or presence of 1 *μ*g/ml LPS for 18 h; then, cell viability was measured by MTT assay. Data are presented as means ± SD of three independent experiments.

The inhibitory effect of TFE on RAW 264.7 cell growth was evaluated by the MTT assay. Cells were incubated with TFE at concentrations of 50, 100, and 200 *μ*g/ml after being pretreated with LPS or medium only. The results showed that cell viabilities (99.3–100.7%) were not significantly (*p* > 0.05) affected by TFE in the experimental concentration range (Figure 3).

#### 3.6.2. Effect of TFE on the Content of COX-2 and NO and on the Genes Expression Levels of *COX-2* and *iNOS*

Previous studies indicated that baicalein can inhibit *COX-2* gene expression in LPS-induced RAW 264.7 cells. Moreover, wogonin and quercetin suppressed NO production in a dose-dependent manner in the same model. In addition, rutin (80 *μ*M) clearly displayed an inhibitory effect on NO production induced by LPS in primary peritoneal macrophages [50, 51]. Furthermore, wogonin significantly inhibited NO production by suppressing iNOS mRNA and protein expression levels [52].

**Figure 4:**
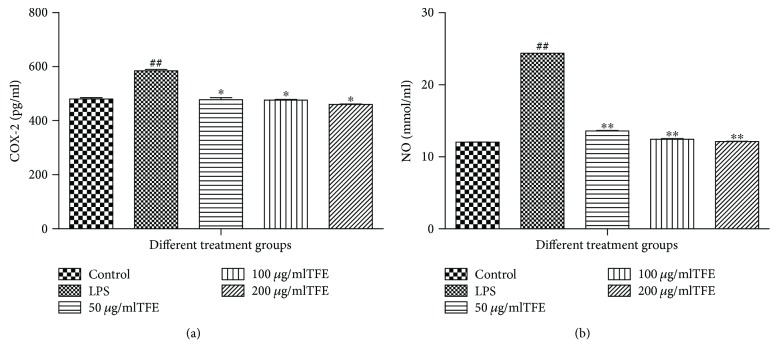
Effects of different concentrations of TFE on LPS induced COX-2 (a) and NO (b) production in RAW 264.7 cells. Cells were pretreated with TFE (50, 100, and 200 *μ*g/ml) for 1 h, followed by LPS (1 *μ*g/ml) stimulation for 18 h. Values represent the mean ± SD of the three independent experiments (^#^^#^compared with the control, ^∗^compared with LPS; ^∗^*p* ≤ 0.05 and ^∗∗/##^*p* ≤ 0.01).

To investigate the anti-inflammatory activities and molecular mechanisms of TFE from *A. theophrasti* leaves, we first examined the ability of TFE to regulate the production of COX-2 and NO and their gene expression levels in response to LPS stimulation. According to the results shown in Figure 4, the concentrations of COX-2 and NO increased very significantly (*p* ≤ 0.01) in the LPS group compared to the control group, and compared with the LPS group, their concentrations significantly decreased in a dose-dependent manner in the experimental groups pretreated with 50, 100, and 200 *μ*g/ml of TFE.

**Figure 5:**
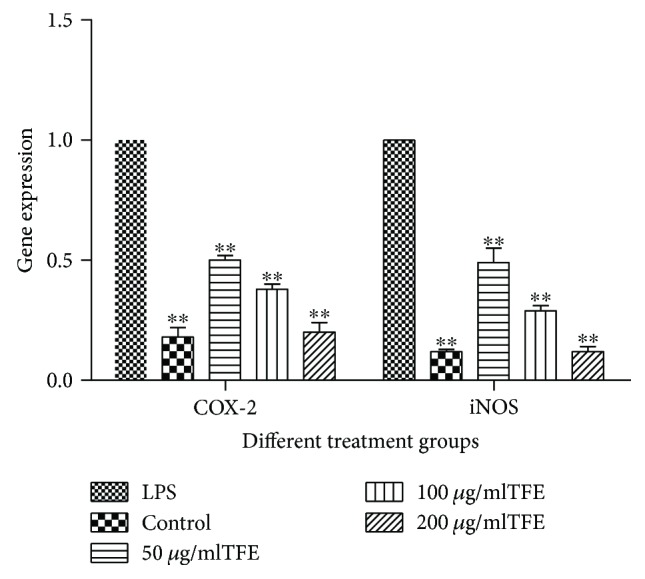
Effects of different concentrations of TFE on LPS induced COX-2 and iNOS genes expression in RAW 264.7 cells. Cells were pretreated with TFE (50, 100, and 200 *μ*g/ml) for 1 h, followed by LPS (1 *μ*g/ml) stimulation for 18 h. Values represent the mean ± SD of the three independent experiments (^∗∗^compared with LPS; ^∗∗/##^*p* ≤ 0.01).

Based on the inhibitory effect of TFE against the production of COX-2 and NO induced by LPS, the regulation of their gene expression was examined in TFE-treated RAW 264.7 cells. As shown in Figure 5, the expression levels of *COX-2* and *iNOS* genes were significantly (*p* ≤ 0.01) decreased in a concentration-dependent manner in the groups treated with 50, 100, and 200 *μ*g/ml of TFE compared with the LPS-treated group. These studies indicated that TFE has a direct inhibitory effect on the production of COX-2 and NO and blocks their gene expression in LPS-induced RAW 264.7 cells. The results are consistent with those reported in the literature.

#### 3.6.3. Effect of TFE on the Cytokine Secretion

In a study by Abdallah et al. [53], the anti-inflammatory activities of amentoflavone, apigenin-7-*O*-*β*-D-glucopyranoside, acacetin-7-*O*-*β*-D-[*α*-L-rhamnosyl(1→6)]3^″^-E-*p*-coumaroyl glucopyranoside and rutin were assessed by measuring the levels of IL-1*β*, IL-6, and TNF-*α* in the supernatant media of human peripheral blood mononuclear cells (PBMCs) stimulated by phytohemagglutinin. The four isolated flavonoid compounds decreased the content of IL-1*β*, IL-6, and TNF-*α* significantly at a concentration of 100 *μ*M [53].

Moreover, Nakamura et al. [54] reported that the production and mRNA expression levels of IL-6 and IL-8 were significantly suppressed by baicalein and wogonin in a study of the effects of baicalin, baicalein, and wogonin on the protein and mRNA expression levels of IL-6 and IL-8 and on NF-*κ*B binding activities induced by IL-1*β* in a human retinal pigment epithelial cell line cells.

**Figure 6:**
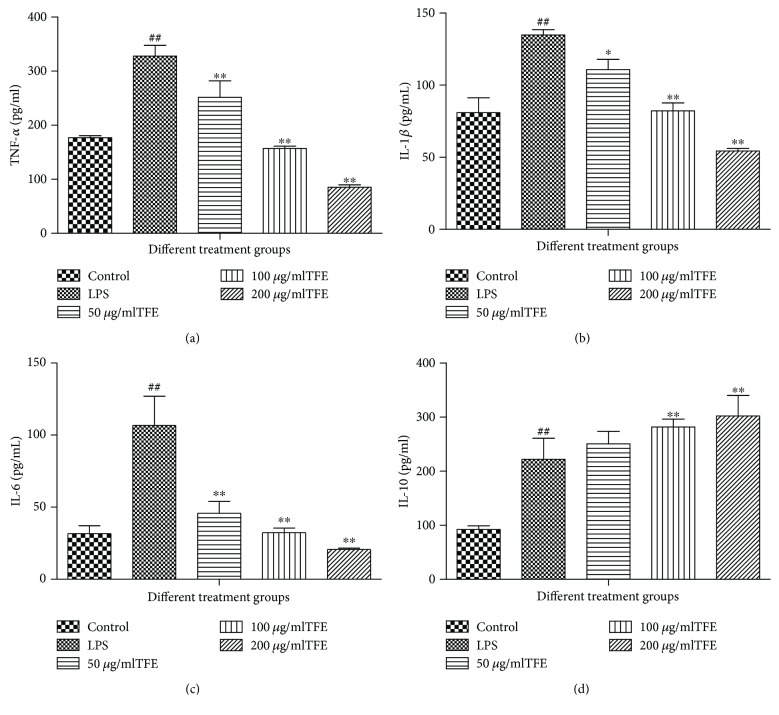
Effect of different concentrations of TFE on secretion of TNF-*α* (a), IL-1*β* (b), IL-6 (c), and IL-10 (d) *in vitro*. The cells were treated with LPS alone or LPS plus different concentrations (50, 100, and 200 *μ*g/ml) of TFE for 18 h. The values represent means ± SD of the three independent experiments (^#^^#^compared with the control, ^∗^compared with LPS; ^∗^*p* ≤ 0.05 and ^∗∗/##^*p* ≤ 0.01).

In the present study, 50, 100, and 200 *μ*g/ml of TFE reduced (*p* ≤ 0.01) the content of IL-1*β*, IL-6, and TNF-*α* and enhanced (*p* ≤ 0.05) the concentration of IL-10 compared to the LPS-treated group (Figure 6). The research suggested that TFE from *A. theophrasti* leaves can suppress the inflammatory response directly by decreasing the production of proinflammatory cytokines and by increasing the content of suppression inflammatory cytokines. The results are consistent with the above literatures.

#### 3.6.4. Effect of TFE on LPS-Induced NF-*κ*B Signaling Pathways Activation

**Figure 7:**
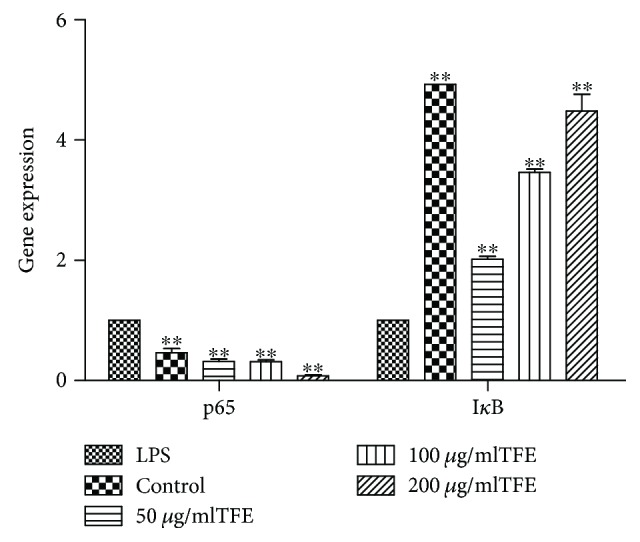
Effects of different concentrations of TFE on the genes expression of p65 and I*κ*B in NF-*κ*B signaling pathway in LPS induced RAW 264.7 cells. Cells were pretreated with TFE (50, 100, and 200 *μ*g/ml) for 1 h, followed by LPS (1 *μ*g/ml) stimulation for 18 h. Values represent the mean ± SD of the three independent experiments (^∗∗^compared with LPS; and ^∗∗/##^*P* ≤ 0.01).

NF-*κ*B signaling pathways are important for regulation of the synthesis and secretion of cytokines and mediators of the inflammatory response [55, 56]. To evaluate whether NF-*κ*B signaling pathways are related to the anti-inflammatory activity of TFE from *A. theophrasti* leaves, RAW 264.7 cells were pretreated with 50, 100, and 200 *μ*g/ml of TFE for 1 h followed by LPS (1 *μ*g/ml) stimulation for 18 h. The levels of expression of *p65* and *IκB* genes were determined by real-time fluorescence quantitative PCR with *β-actin* as a reference gene. As shown in Figure 7, we found that mRNA expression of the *p65* gene increased significantly (*p* ≤ 0.01) and that *IκB* gene expression decreased significantly (*p* ≤ 0.01) in the LPS-induced RAW 264.7 cells compared to the control group. The data indicate that the experimental model was established successfully. Moreover, TFE from *A. theophrasti* leaves significantly suppresses mRNA expression of the *p65* gene (*p* ≤ 0.01) and significantly increases *IκB* gene expression (*p* ≤ 0.01) compared with the LPS treatment group.

**Figure 8:**
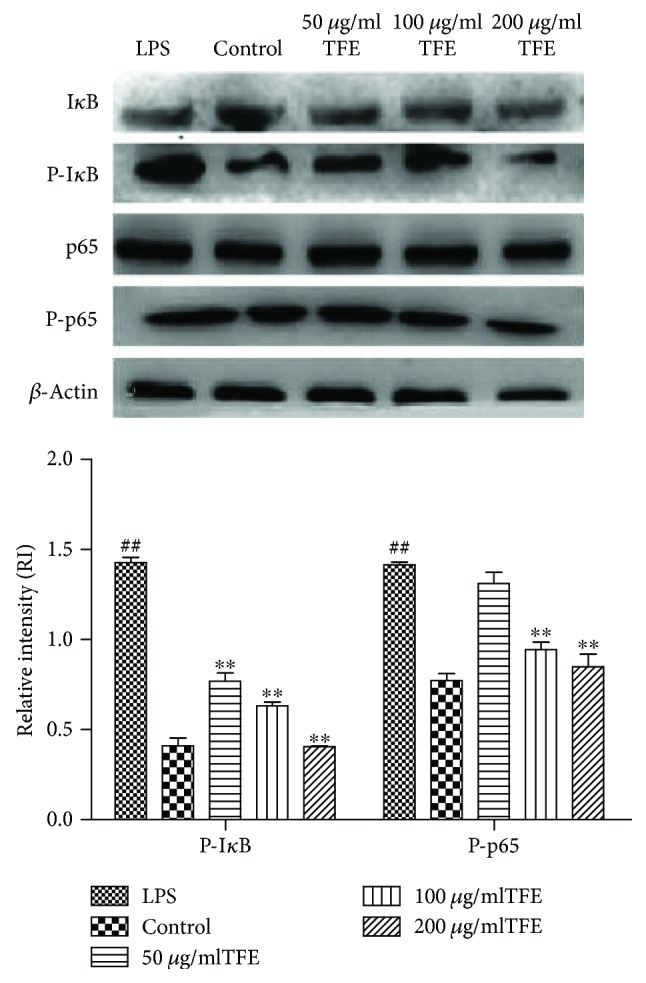
Effects of different concentrations of TFE on the proteins expression of P-I*κ*B and P-p65 in NF-*κ*B signaling pathway in LPS induced RAW 264.7 cells. Cells were pretreated with TFE (50, 100, and 200 *μ*g/ml) for 1 h, followed by LPS (1 *μ*g/ml) stimulation for 18 h. The proteins expression were determined by Western blot analysis, and *β*-actin served as an internal control. Values represent the mean ± SD of the three independent experiments (^#^^#^compared with the control, ^∗∗^compared with LPS; ^∗∗/##^*p* ≤ 0.01).

The phosphorylation levels of two important NF-*κ*B signaling molecules (I*κ*B and p65) were analyzed by Western blot for further clarification of the anti-inflammatory effects of TFE on NF-*κ*B signaling pathways. As shown in Figure 8, the phosphorylation levels of I*κ*B and p65 rose rapidly with treatment by LPS compared with controls. Furthermore, Figure 8 also shows that the degradation of I*κ*B and phosphorylation levels of p65 induced by LPS were inhibited by TFE in a dose-dependent manner. In addition, the expression of p-I*κ*B was reduced significantly by treatment with 50, 100, and 200 *μ*g/ml of TFE. These results indicated that TFE possibly exerts its anti-inflammatory effects by the NF-*κ*B signaling pathway in LPS-activated RAW 264.7 cells.

#### 3.6.5. Effect of TFE on LPS-Induced MAPK Signaling Pathway Activation

**Figure 9:**
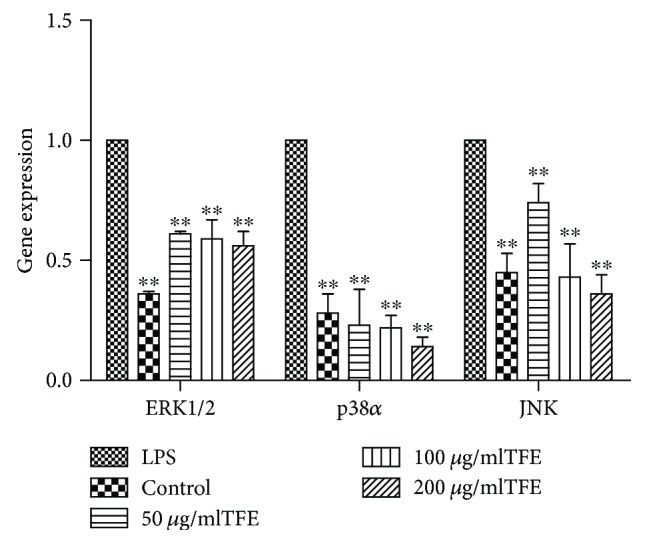
Effects of different concentrations of TFE on the genes expression of ERK1/2, p38*α*, and JNK in MAPK signaling pathway in LPS induced RAW 264.7 cells. Cells were pretreated with TFE (50, 100, and 200 *μ*g/ml) for 1 h, followed by LPS (1 *μ*g/ml) stimulation for 18 h. Values represent the mean ± SD of the three independent experiments (^∗^compared with LPS; ^∗∗/##^*p* ≤ 0.01).

To better understand the mechanisms underlying the anti-inflammatory action, we assessed the effect of TFE from *A. theophrasti* leaves on the MAPK signaling pathways, which are other important signaling pathways for modulating inflammation [57, 58]. The inhibitory effect of TFE on inflammation was investigated by studying the MAPK signaling pathways. The expression levels of *ERK1/2*, *p38*, and *JNK* genes were examined by real-time fluorescence quantitative PCR, and *β-actin* was used as an internal control. As shown in Figure 9, the expression levels of *ERK1/2*, *p38*, and *JNK* mRNA increased significantly after stimulation by LPS (1 *μ*g/ml) compared to those of the control group; they were all significantly suppressed by 50, 100, and 200 *μ*g/ml of TFE compared with those of the LPS treatment group.

**Figure 10:**
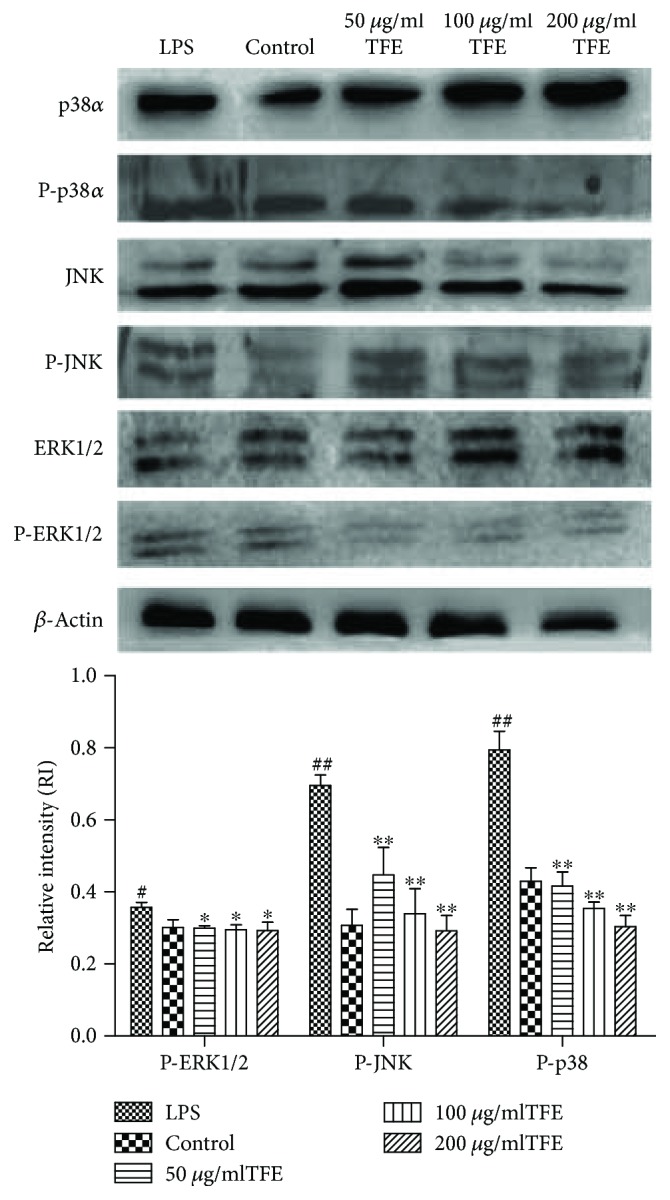
Effects of different concentrations of TFE on the proteins expression of P-p38*α*, P-JNK, and P-ERK1/2 in MAPK signaling pathway in LPS induced RAW 264.7 cells. Cells were pretreated with TFE (50, 100, and 200 *μ*g/ml) for 1 h, followed by LPS (1 *μ*g/ml) stimulation for 18 h. The proteins expression were determined by Western blot analysis, and *β*-actin served as an internal control. Values represent the mean ± SD of the three independent experiments (^#^compared with the control, ^∗^compared with LPS, ^∗/#^*P* ≤ 0.05, and ^∗∗/##^*P* ≤ 0.01).

To evaluate whether the inhibitory action of TFE on the secretion of inflammatory mediators and cytokines is mediated through the MAPK signaling pathways, the influence of TFE on LPS-stimulated phosphorylation of ERK1/2, JNK, and p38MAPK in RAW 264.7 cells was examined by Western blot analysis with phosphospecific antibodies. Cells were pretreated with 50, 100, and 200 *μ*g/ml of TFE for 1 h and then stimulated with 1 *μ*g/ml of LPS for 18 h. As shown in Figure 10, the phosphorylation levels of three important MAPK signaling molecules (ERK1/2, JNK, and p38MAPK) were significantly inhibited by different concentrations of TFE. These experimental results indicated that the secretion of TNF-*α*, IL-1*β*, and IL-6 could be inhibited by TFE by blocking the activation of MAPK signaling pathways.

**Figure 11:**
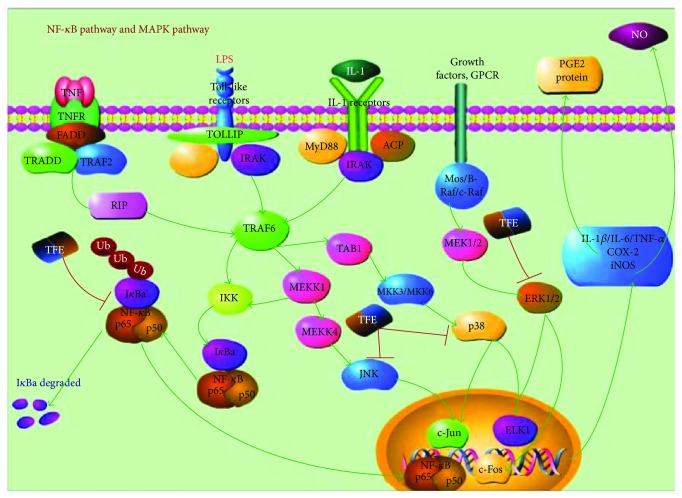
The proposed role of TFE on LPS-induced production of proinflammatory mediators and cytokines in RAW 264.7 cells.

Briefly, NF-*κ*B and MAPK signaling pathways are considered closely related to the regulation of inflammation by influencing the secretion of proinflammatory cytokines and mediators. LPS, as an inducer, can activate macrophages by stimulating toll-like receptor 4 (TLR4) and triggering NF-*κ*B and MAPK selectively. MyD88 and other small molecule proteins were enriched in toll-like receptors after LPS treatment. In addition, tumor necrosis factor receptor-associated factor 6 (TRAF), a ubiquitin ligase, will produce a series of related enzymes through ubiquitination reactions. In the NF-*κ*B signaling pathways, I*κ*B kinase (IKK) can regulate the expression of the downstream genes by degrading I*κ*B, translocating the p65-p50 dimer into the nucleus and increasing DNA binding sites. As shown in Figure 11, TFE plays an important role in anti-inflammatory activities and not only increases I*κ*B gene expression effectively and increases the p65-p50 dimer but also reduces expression of the *p65 gene*. In the MAPK signaling pathways, TFE clearly displayed anti-inflammation activity via inhibition of phosphorylation of ERK1/2, JNK, and p38MAPK and prevention of their degradation into small molecular fragments and of combination with the DNA-targeted genes.

## 4. Conclusion

In conclusion, this study demonstrated for the first time the specific extraction process, component analysis, and *in vitro* antioxidant, antibacterial, and anti-inflammatory activities of TFE from *A. theophrasti* leaves in LPS-stimulated RAW 264.7 cells. The extraction procedure of TFE was optimized by single-factor experimentation and RSM with three variables and three levels, and the extraction conditions were as follows: extraction time 30 min; concentration of ethanol solution 42.28%; and ratio of solvent to material 30 : 1 ml/g. Seven major components of TFE were identified by HPLC-DAD-ESI-MS^n^. In addition, TFE displayed significant antioxidant, antibacterial, and anti-inflammatory activities. It is worth mentioning that TFE exerts anti-inflammatory activities by activation of both NF-*κ*B and MAPK signaling pathways. However, the effects of TFE on other signaling pathways, such as TLR4 and AP-1, will be investigated in future studies. In addition, future studies will focus on providing additional mechanistic evidence for the effects of TFE on signaling pathways *in vivo*. Especially noteworthy, TFE from *A. theophrasti* leaves can be regarded as a new, healthy, and natural drug or additive that is associated with antioxidation and antibacterial and anti-inflammatory activities for the pharmaceutical and food industries.

## Figures and Tables

**Table 1:** The primer sequences of fluorescence qRT-PCR.

Genes	Primer direction	Primer sequences (5′–3′)	Reannealing temperature (°C)	Product size (bp)
*COX-2*	Forward	AGCCAGGCAGCAAATCCTT	60	169
	Reverse	GGGTGGGCTTCAGCAGTAAT		

*iNOS*	Forward	GGTGAAGGGACTGAGCTGTT	60	103
	Reverse	ACGTTCTCCGTTCTCTTGCAG		

*IκB*	Forward	GCCATCCCAGGCAGTATCTA	60	106
	Reverse	TTCCAAGACCAGACCTCCAG		

*p65*	Forward	GACCTGGAGCAAGCCATTAG	60	123
	Reverse	CACTGTCACCTGGAAGCAGA		

*p38α*	Forward	CTATGGCTCGGTGTGTGCT	60	115
	Reverse	GACGCAACTCTCGGTAGGTC		

*JNK*	Forward	TGTTCCCCGATGTGCTTTT	59.7	147
	Reverse	CGTTGATGTATGGGTGCTG		

*ERK1/2*	Forward	GCGGCTGAAGGAGTTGAT	60	119
	Reverse	CAGGTAGGAGCAGGACCAGA		

*β-Actin*	Forward	GTGCTATGTTGCTCTAGACTTCG	60	174
	Reverse	ATGCCACAGGATTCCATACC		

**Table 2:** Independent variables and the levels used in the Box-Behnken design.

Independent variables	Levels		
	−1	0	1
Extraction time (*A*, min)	20	25	30
Concentration of ethanol solution (*B*, %)	30	40	50
Ratio of solvent to material (*C*, ml/g)	20	25	30

**Table 3:** The Box-Behnken design matrix and response values for TFE yields.

Run	Variable levels			Response 1 (R1)
	A	B	C	
1	25	40	25	1.09
2	20	30	25	1.04
3	20	40	20	1.02
4	30	30	25	1.02
5	25	30	30	1.21
6	30	40	20	1.17
7	30	40	30	1.29
8	25	40	25	1.08
9	20	40	30	1.16
10	25	40	25	1.09
11	25	50	30	1.09
12	20	50	25	0.85
13	25	40	25	1.08
14	25	50	20	1.07
15	30	50	25	1.18
16	25	30	20	1.00
17	25	40	25	1.09

**Table 4:** ANOVA for the response surface quadratic model of TFE yields.

Source	Sum of squares	DF	Mean square	*F* value	*p* value, prob > *F*	Significant
Model	0.15	9	0.017	299.31	<0.0001	Significant
*A*	0.044	1	0.044	771.11	<0.0001	^∗∗^
*B*	0.0008	1	0.0008	14.18	0.0070	^∗∗^
*C*	0.030	1	0.030	531.87	<0.0001	^∗∗^
*AB*	0.031	1	0.031	542.72	<0.0001	^∗∗^
*AC*	0.0001	1	0.0001	1.77	0.2248	
*BC*	0.009025	1	0.009025	159.94	<0.0001	^∗∗^
*A* ^2^	0.00001684	1	0.00001684	0.30	0.6018	
*B* ^2^	0.018	1	0.018	320.13	<0.0001	^∗∗^
*C* ^2^	0.022	1	0.022	386.81	<0.0001	^∗∗^
Residual	0.000395	7	0.00005643			
Lack of fit	0.000275	3	0.00009167	3.06	0.1544	Not significant
Pure error	0.00012	4	0.00003			
Cor total	0.15	16				
*R* ^2^	0.9974					
Adj *R*^2^	0.9941					
Pred *R*^2^	0.9699					
Adeq precision	76.154					
CV%	0.69					

^∗∗^ *p* ≤ 0.01.

**Table 5:** TFC (%) and IC_50_ values for evaluating antioxidant assays and EC_50_ values for reducing power of TFE from *A. theophrasti* leaves.

Number	TFC (%)	IC_50 ABTS_ (*μ*g/ml)	IC_50 DPPH_ (*μ*g/ml)	EC_50 FRAP_ (mmol Fe^2+^/0.1 *μ*g/ml)
Aug. sample	1.29 ± 0.02	3.10	8.96	0.33
Sep. sample	1.31 ± 0.01	2.95	8.41	0.41
Oct. sample	0.83 ± 0.02	5.31	14.41	0.23
BHT	—	2.25	8.11	0.14

IC_50_: inhibition concentration 50%.

**Table 6:** The major compounds in TFE identified by HPLC-DAD-ESI-MS^n^ analysis.

Peak	Rt	[M-H]^−^	MS/MS [M − H]^−^	[M + H]^+^	MS/MS [M + H]^+^	Calculated mass	Formula	Proposed molecule	References^b^
1^a^	30.1	609	463,301	611	465,303	610	C_27_H_30_O_16_	Rutin	[24]
2	31.5	463	301	465	303	464	C_21_H_20_O_12_	Quercetin-7-*O*-*β*-glucoside	[49]
3	32.3	593	447,285	595	449,287	594	C_27_H_30_O_15_	Kaempferol 3-O-*α*-rhamnopyranosyl(1→6)-*β*-glucopyranoside	[49]
4	32.5	285	—	287	—	286	C_15_H_10_O_6_	Luteolin	[24]
5	41.3	593	447,285	595	449,287	594	C_27_H_30_O_15_	Apigenin-7-O-*β*-diglucoside	[46]
6	42.1	593	447,285	595	449,287	594	C_27_H_30_O_15_	Poncirin	[47]
7	48.7	593	447,285	595	449,287	594	C_27_H_30_O_15_	Tiliroside	[48]

^a^Peak number as in Figure 2. ^b^The reference column refers to previous reports on metabolites in different plants.

**Table 7:** Correlation coefficients between assays.

	ABTS	DPPH	FRAP	TFC (%)
ABTS	1	1.000^∗^	−0.920	−1.000^∗^
DPPH	—	1	−0.930	−0.999^∗^
FRAP	—	—	1	0.912
TFC (%)	—	—	—	1

^∗^Significant at *p* ≤ 0.05.

**Table 8:** MIC values of TFE from *A. theophrasti* leaves against pathogenic bacteria (mean ± SD; *n* = 3).

Sample	*Escherichia coli* (ATCC 25922)	*Salmonella* (ATCC 51812)	*Staphylococcus aureus* (ATCC 25923)	*Streptococcus* (ATCC 49619)
Gentamicin (*μ*g/ml)	2.00 ± 0.02	0.50 ± 0.01	0.50 ± 0.01	1.00 ± 0.01
TFE (g crude drug/ml)	1.02 ± 0.04	0.51 ± 0.02	0.06 ± 0.01	0.26 ± 0.01
